# *Fusarium*: Mycotoxins, Taxonomy, Pathogenicity

**DOI:** 10.3390/microorganisms8091404

**Published:** 2020-09-12

**Authors:** Łukasz Stępień

**Affiliations:** Department of Pathogen Genetics and Plant Resistance, Institute of Plant Genetics, Polish Academy of Sciences, Strzeszyńska 34, 60-479 Poznań, Poland; lste@igr.poznan.pl; Tel.: +48-61-655-0286

It has been over 200 years since *Fusarium* pathogens were described for the first time, and they are still in the spotlight of researchers worldwide, mostly due to their mycotoxigenic abilities and subsequent introduction of harmful metabolites into the food chain. The accelerating climatic changes result in pathogen populations and chemotype shifts all around the world, thus raising the demand for continuous studies of factors that affect virulence, disease severity and mycotoxin accumulation in plant tissues. This Special Issue summarizes recent advances in the field of *Fusarium* genetics, biology and toxicology.

An emphasis was bestowed upon trichothecene-producing species. *Fusarium graminearum* and *F. culmorum* are the prevailing deoxynivalenol (DON) producers. Inoculation of wheat with the mixture of isolates resulted in lower disease incidence than in the case of the single aggressive isolate, showing a considerable level of competition between the genotypes during the colonization of the plant [1]. A similar observation can be made when a pathogenic *Fusarium* strain is co-inoculated with the non-pathogenic endophytic strain. The endophyte decreases the efficiency of root infection by the pathogen, reducing the colonization and increasing the plant resistance [2]. The non-pathogenic strains may also alter the methylation patterns of the genes related to the pathogenesis response of the host plants, which results in an altered reaction to the pathogen encounter [3].

An organic farming system may also contribute to the increased incidence of *Fusarium* pathogens in the grain. The seed quality is lower, however, it is not clear if growing wheat without chemical protection results in the increased accumulation of mycotoxins when compared to the conventional farming system [4]. After the harvest, the stored grain may also be a source of mycotoxins, particularly when the storage conditions are favourable for fungal growth and proliferation. Water activity and spatial location of the inoculum are also important for grain colonization [5].

Environmental factors are known to play significant roles in disease progression. The precise time of inoculation in relation to the flowering is essential to define the “susceptibility window” for effective infection [6]. Moreover, it influences the levels of *Fusarium* metabolites and mycotoxins produced and accumulated in the grain as the infection proceeds [7]. The influence of weather on the contamination of plant material with *Fusarium* species and mycotoxins is even more obvious when the year-to-year variation is considered. A correlation is often found between weather conditions and the occurrence of the specific mycotoxin groups, e.g., fumonisins in maize [8]. Another factor influencing the virulence of *Fusarium* pathogens is light. Recent studies revealed the existence of a photo-sensor component which is not only responsible for the ecological adaptation of the pathogen, but also allows for the light regulation of the virulence expression [9]. The *Fusarium graminearum* virulence factors already discovered using modern New Generation Sequencing (NGS) and proteomic techniques were comprehensively reviewed [10], which will likely boost the research of other pathogenic species.

The mycotoxigenic abilities of the *Fusarium* populations have been widely studied for many years. In general, there is no correlation between the efficiency of the metabolite synthesis and geographical origin of the strains studied. Nevertheless, there are reports of significant differences in the toxin production that occur despite the genetic uniformity of the population over a large area [11]. The chemotypes emerging in unexplored geographic areas could be a serious threat. Constant monitoring of the main mycotoxin groups should be performed, as new analogues of well-known compounds can have similar or higher activities against plant, animal and human cells, as was discovered for the NX-3 trichothecene [12]. The ability of plant-pathogenic *Fusarium* species to infect humans has also been reported [13]. This confirms the enormous adaptability of the genus members in searching for new ecological niches.

In conclusion, constant progress in *Fusarium* research can be observed and is expected in the future in all areas of fungal biology, pathology and toxicology, especially with the aid of modern techniques, deployed to uncover the mechanisms of secondary metabolism regulation, interspecific molecular communication and virulence modulation by external and internal agents.
